# Intra-articular knee haemangioma originating from the anterior cruciate ligament: a case report

**DOI:** 10.1186/1752-1947-2-254

**Published:** 2008-07-28

**Authors:** Mathaios Tzurbakis, George Mouzopoulos, Emmanouil Morakis, Gerogios Nikolaras, Ioannis Georgilas

**Affiliations:** 1 1st Orthopaedic Department of Evangelismos Hospital, Athens, Greece

## Abstract

**Introduction:**

Synovial haemangioma is a rare intra-articular benign tumour, which may arise from any synovium-lined surface, but particularly in the knee joint. Synovial haemangioma originating from the anterior cruciate ligament has not been reported previously.

**Case presentation:**

A 34-year-old man presented with a history of intermittent knee pain, locking and swelling.

**Conclusion:**

Knee intra-articular haemangioma, a very rare benign tumour, is often misdiagnosed. Magnetic resonance imaging is effective in detecting this lesion and should be performed in cases of persistent knee swelling and pain.

## Introduction

Synovial haemangioma is a rare cause of knee pathology which, when undiagnosed, can lead to significant morbidity. Fewer than 200 synovial haemangiomas have been reported previously [1] and, to the best of the authors' knowledge, an intra-articular haemangioma originating from the anterior cruciate ligament has never been reported. We report such a case, describe its clinical and imaging findings and discuss appropriate management.

## Case presentation

A 34-year-old man presented with a history of almost 5 years of symptoms in his right knee. He reported injuring his knee twice after falling 3 years ago, after which he noted intermittent pain whenever he twisted the knee, particularly into external rotation. He also mentioned that there was occasionally locking of his knee.

Physical examination showed normal knee alignment, no joint line tenderness, swelling adjacent to the medial side of the patella and a small amount of fluid in the knee joint. There was also slight muscle atrophy in the affected thigh, with a full range of motion and a great difficulty walking downhill and descending a staircase. Collateral ligaments were intact and McMurray's test was negative.

**Figure 1:**
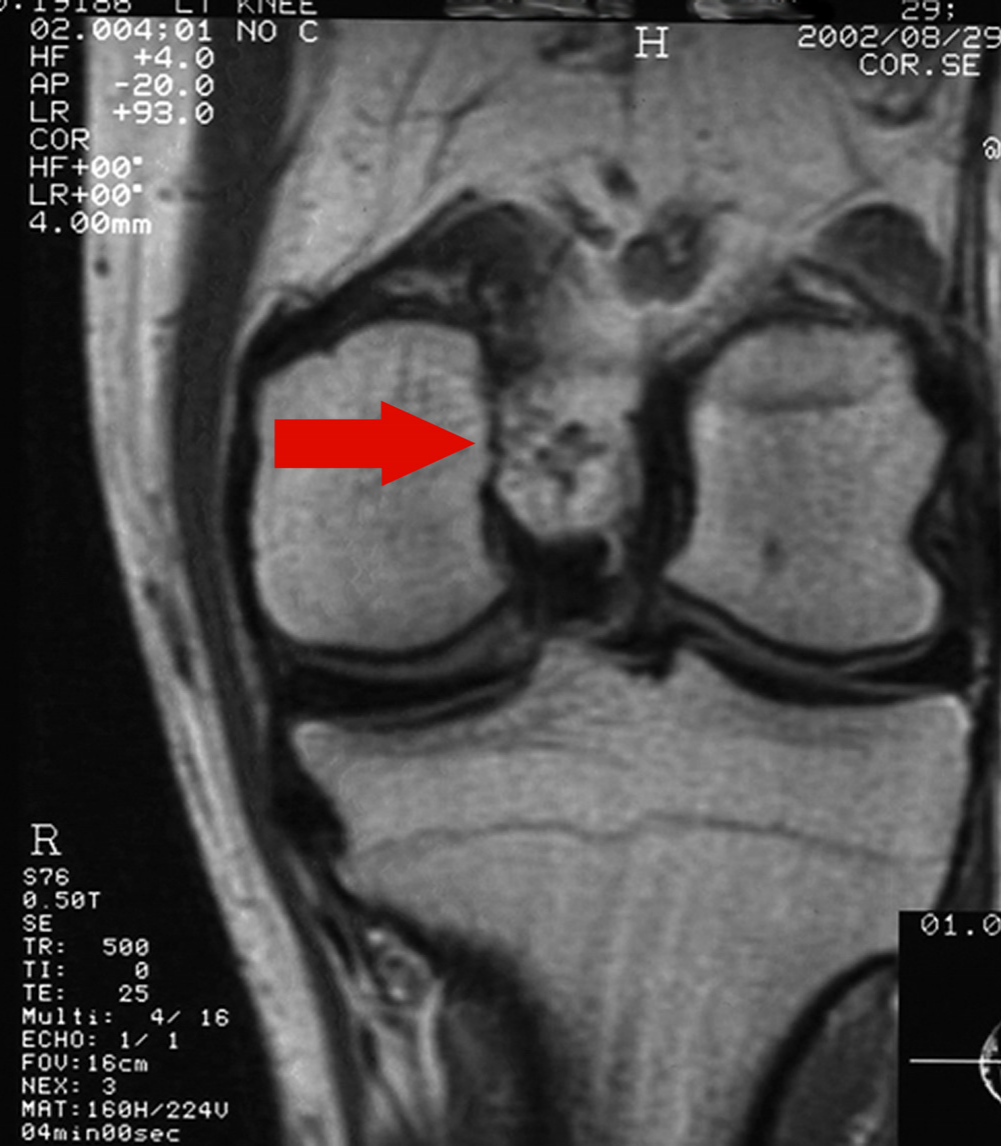
Magnetic resonance imaging showing a haemangioma located in the femoral inter-condylar notch.

Plain X-rays of the knee were normal. A magnetic resonance imaging (MRI) scan showed a multilobulated tumour in the femoral inter-condylar notch cranially to the anterior cruciate ligament, measuring 15 mm in diameter (Figure 1). On T2-weighted images, the tumour was hyperintense, and with lower signal intensity to muscle on T1-weighted sequences.

**Figure 2:**
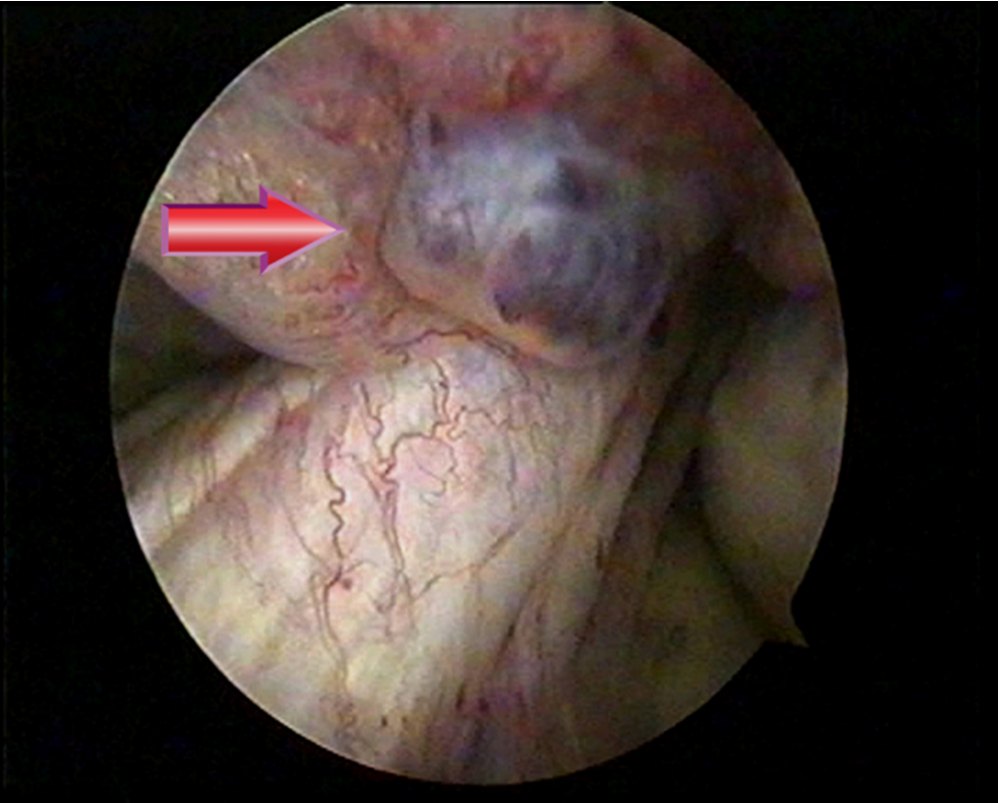
Arthroscopy showing the haemangioma originating from the anterior cruciate ligament.

At knee arthroscopy, a 1.5 × 2 cm cherry-red villous nodular synovial mass was found in the inter-condylar notch, originating from the anterior cruciate ligament. The mass was removed successfully (Figure 2). Histopathological analysis of the specimen showed a lobular proliferation of capillary sized vascular channels consistent with a synovial haemangioma.

On follow-up 2 weeks after surgery the patient was pain-free, with no effusion and with a full range of motion of the knee. At 4-year follow-up no recurrence was detected and the patient remains symptom-free.

## Discussion

Synovial haemangioma is a rare intra-articular benign tumour first described by Bouchut in 1856 (see [2]). It is thought to be a vascular malformation which arises from any synovium-lined surface, rather that a true neoplasm [3]. In 97% of cases, it is located in the knee, but it has also been reported in other joints including the elbow, ankle and wrist [4]. Synovial haemangioma originating from the anterior cruciate ligament has never been reported previously.

Typically the patient is a child or a young adult who presents with a non-traumatic recurrently swollen tender knee and intermittent knee pain [5,6]. Devaney et al. examined 20 patients in their study and reported that the symptoms are usually pain and swelling (31%), pain alone (31%), painless mass (31%) and recurrent intra-articular haemorrhage (5%) [7]. Other symptoms include a limited range of motion or recurrent episodes of locking knee. Knee trauma can lead to bleeding of the haemangioma with haemarthrosis and swelling of the tumour itself. Signs are often minimal but may include atrophy of the quadriceps muscle, movable tendon mass, joint effusion and restricted range of movement [8].

As the majority of patients present with non-specific symptoms and signs, diagnosis is frequently delayed for several years. The non-specific symptoms occur in childhood, which adds to the difficulty of making a diagnosis, and physicians therefore tend to misdiagnose it as a growing pain [2]. When the lesion is located adjacent to the medial side of the patella, physicians are likely to make an incorrect diagnosis of medial shelf syndrome. Clinical findings may lead to the diagnosis of a meniscal tear, especially in cases with a history of trauma [9].

Diagnostic delay is problematic in terms of loss of function. However, the lesions can also increase in size and infiltrate surrounding tissues, making excision more difficult and resulting in invasion of adjacent bone. Furthermore, recurrent haemarthrosis can lead to an arthropathy similar to that seen in haemophilia [10].

MRI is important in detecting intra-articular haemangiomas and it seems to be more accurate than computed tomography in defining the size and extent of the tumour. Furthermore, it shows any existing chondral degeneration [4]. Arthroscopy may show the tumour if it is in a visible region.

Arthroscopic total excision must be the choice of treatment if the tumour is intra-articular, pedunculated, localized and manageable in size [1,2]. Open local surgical resection with partial or total synovectomy is indicated for the haemangiomas that are widespread but situated in one compartment, and in cases of excessive bleeding caused by arthroscopy [10]. Pre-operative embolism may be helpful in diffuse lesions that have large arterial feeding vessels [1]. Total knee arthroplasty is the treatment of choice in cases of high-grade degeneration of the chondral tissues.

## Conclusion

Knee intra-articular haemangioma is a very rare benign tumour which is often misdiagnosed. MRI is effective in detecting this lesion and should be performed in cases of persistent swelling knee and pain.

## Abbreviations

MRI: Magnetic resonance imaging.

## Competing interests

The authors declare that they have no competing interests.

## Authors' contributions

MT performed the arthroscopy and contributed to the writing of the manuscript. GM analyzed and interpreted the patient data and contributed to the writing of the manuscript. EM, GN and IG contributed to the writing of the manuscript. All authors read and approved the final manuscript.

## Consent

Written informed consent was obtained from the patient for publication of this case report and accompanying images. A copy of the written consent is available for review by the Editor-in-Chief of this journal.
